# Rehabilitative Support for Persons with Dementia and Their Families to Acquire Self-Management Attitude and Improve Social Cognition and Sense of Cognitive Empathy

**DOI:** 10.3390/geriatrics4010026

**Published:** 2019-02-25

**Authors:** Yohko Maki, Hideyuki Hattori

**Affiliations:** National Center for Geriatrics and Gerontology 7-430, Morioka, Obu, Aichi 474-8511, Japan

**Keywords:** dementia, quality of life, rehabilitation, occupational therapy, pro-social relationship, social reserve, cognitive empathy

## Abstract

People with dementia are often inevitably confronted with various difficulties with social interaction and communication, which is a core problem that can be improved with rehabilitative support, thus improving their quality of life. The authors propose rehabilitative support using communication via activities; co-beneficial relationship-based rehabilitation, which emphasizes the following 3 points: support for people with dementia to improve social reserves, which is the ability to overcome the decline in social cognition; support for family members for improving cognitive empathy, which is the ability to analyze the background of others’ behaviors and speech; and the involvement of the practitioner to supervise and empower them. The process of intervention is as follows: (1) selecting activities for collaboration; (2) sharing information on their current situation including declined abilities; (3) enhancing cognitive empathy through dementia education; (4) designing the intervention measures together; and (5) practice and feedback. Living with dementia involves a continuous process of coping with various challenges in daily living, however, the process of effectively managing these challenges is one of the ways to improve the quality of life of people with dementia and their family members.

## 1. Dementia and Social Interaction

Dementia is a disease that affects social relationships. We propose a method of rehabilitative support for people with dementia and their family members to empower them with a self-management attitude, to live in harmony, with a superior quality of life (QOL). In this manuscript, the term “social participation” has been used to refer to all the human relationships including the relationship within family members, which is one of the most important social relationships. Therefore, until the final stage of dementia, each person has various social relationships with others.

### 1.1. Social Cognition in Dementia 

The Diagnostic and Statistical Manual of Mental Disorders, Fifth Edition (DSM-5) defines dementia (termed as a major neurocognitive disorder) as follows: “There is evidence of substantial cognitive decline from a previous level of performance in one or more of the domains; the cognitive deficits are sufficient to interfere with independence (i.e., requiring minimal assistance with instrumental activities of daily living).” That is, dementia is a disease that affects independent living, thus, people with dementia inevitably require support for daily living. Furthermore, DSM-5 proposes 6 domains of cognitive functions: complex attention, executive function, learning and memory, language, perceptual-motor function and social cognition, which has been recently recognized as an independent cognitive domain [1]. 

Social cognition refers to the ability “to know oneself and to know others.” The representative ability of the latter involves the theory of mind (ToM) reasoning, that is “the ability to infer the representational mental state of another individual, such as a belief or intention” [2] and ToM reasoning has been reported in a meta-analysis to be declined in early stage of Alzheimer’s disease dementia (ADD) [3,4], as well as in our study cases [5,6]. The former in the context of dementia is self-awareness about behavioral and/or cognitive deficits and these deficits are recognized as one of the characteristic symptoms of ADD [7,8,9].

### 1.2. Difficulties in Social Interaction Due to Decline in Social Cognition and Other Cognitive Function

It should be noted that the decline in cognitive functions other than social cognition also affects the abilities required in decision making for daily living, assessment of situation and verbal communication, therefore, such difficulties and miscommunication can cause hindrance in social interaction, despite the inability to live without the support of the others. Therefore, improving social abilities for better relationships can be one of the primary factors for a better life-with-dementia. Consequently, rehabilitative support to improve social abilities can provide meaningful support for people with dementia and their families.

## 2. Rehabilitation for Person with Dementia to Improve Social Health

### 2.1. Social Health in Dementia

Social health is characterized by three dimensions: (i) the ability to achieve full potential and fulfil obligations; (ii) the ability to manage life with some degree of independence despite a medical condition; and (iii) the ability to participate in social activities including work [10]. This statement holds true for people with dementia at an early stage [11]. Dementia directly affects the ability to retain some roles in social activities, however, it is important to maintain a proactive intention and the will to participate and contribute to the society in their own capacities. Here, both psychological and instrumental rehabilitative support is desirable; with respect to the psychological aspect, as living with dementia is fraught with various difficulties, encouragement is beneficial to maintain proactive positive intentions, while for instrumental support, empowerment is desirable to execute the residual abilities and to improve social well-being.

### 2.2. Dementia Rehabilitation from the Perspective of International Classification of Functioning, Disability and Health

Rehabilitation aims not only to restore functions but also to reconstruct life with disability, including relationships with others. International Classification of Functioning, Disability and Health (ICF), proposed at the World Health Organization (WHO) General Assembly in 2001 [12], is considered as a basic concept of rehabilitation aimed at improving social health. ICF divides health condition into body functions and structures, activity level and participation level and introduces contextual factors, such as personal and environmental factors, each of which can be both promoting and inhibiting factors. ICF is an interactive model; even if physical and mental functions are the same, the state of activity and participation levels can be different in interaction with different contextual factors, most important of which is social relationship environmental factors including family carers.

The concept of ICF is to provide support to live well with disability and impairment across the life-course [12], which is valuable especially for rehabilitation in dementia, as there is no cure for dementia and people with dementia and their family members, are inevitably confronted with progressive dementia conditions. The desirable goal of rehabilitation is to establish a social participation level, since the ability to maintain independence is compromised in people with dementia, these people inevitably have to live in association with others.

Another characteristic of ICF is the neutral perspective in evaluation of the positive aspects of strength, residual functions, as well as deficits. Thus, focusing on residual functions is also in line with the concept of ICF.

### 2.3. Occupational Therapy for People with Dementia

Among the rehabilitation therapies, occupational therapy (OT), in particular, emphasizes the importance of social relationships and participation in its practice. The American Occupational Therapy Association (AOTA) defines 8 domains of OT practice: activities of daily living, instrumental activities of daily living, rest and sleep, education, play, leisure and social participation [13] and the effectiveness of OT practice to enhance social participation has been reported in older people [14,15,16]. Furthermore, AOTA has identified “Productive Aging” as a key practice area in the 21st century to meet the needs of a rapidly aging society [17]. As promoting social proactive participation of individuals with disabilities is one of the main concepts of ICF, OT aims to provide specific social participation support for those who need special care and consideration (including people with dementia). To maintain social participation for as long as possible, the practitioners focus on the individual’s residual abilities and emphasize modification of the environment, including human environment, among which, the relationship with family members is one of the most important factors.

## 3. Proposal of Rehabilitation Support Using Communication via Activities: Co-Beneficial Relationship-Based Rehabilitation

As dementia affects independent living, inevitably, the relationship with others including family members is changed and living with dementia constantly requires rebuilding the relationship. If people with dementia complain to their family members directly and vice versa, it may result in dispute. However, it can be fruitful to discuss with each other, while including a third party or face each other through activities (using activity as an intermediary). Inclusion of a third party (practitioner) and the use of activity are fundamental ideas of OT. For example, the home-based program of “The Tailored Activity Program (TAP)” proposed by Gitlin also aims at promoting mutual understanding by sharing activities supervised by a practitioner, which reported a reduction in behavioral symptoms and caregiver burden and an improvement in Quality of Living (QOL), in both, the people with dementia and the family carers [18,19,20,21].

Co-beneficial relationship-based rehabilitation is an intervention to empower people with dementia and their family members with a self-management attitude to live in collaboration. For the people with dementia, as the disease deprives them of independent living, it is all the more important to maintain social participation and support from others; thus, the intervention emphasizes to improve their social reserve [22]. For their family members, the intervention aims at improving cognitive empathy [23]. (‘social reserve’ and ‘cognitive empathy’ are explained in a later section.) In this support, the practitioner supervises and empowers the people with dementia and their family members as a third party, instead of direct instructions. To manage the difficulties they face, one after another, in their everyday lives, it is important to acquire the ability to independently devise strategies, even though this may be a prolonged effort. In due course, their life-with-dementia may work out to be meaningful and worth living.

### 3.1. Rehabilitation Support for People with Dementia: Social Reserves

Social reserve is proposed to overcome the decline in social cognition [22], which tends to deteriorate in early stages of ADD. In our clinical cases, we have observed an individual with dementia at an advanced stage who showed high social adaptability and therefore, support to improve the social reserve may be empirically possible. As dementia progresses, it could be difficult for the people with dementia to presume that others have different thoughts and feelings than theirs due to a decline in ToM reasoning, while the authors have observed cases with superfluous social abilities; they seemed to consciously attempt to maintain good relationship with others even at advanced stages. The intervention method for enhancing social reserves has not yet been established, however, empirically, the social abilities could be maintained by acquiring the habit to consciously appreciate human relationships from early stages of dementia.

### 3.2. Rehabilitation Support for the Family Members of People with Dementia: Cognitive Empathy

Empathy has an emotional aspect in which the mind is moved naturally and a cognitive aspect, which is the ability to analyze the background of others’ behavior and speech and to envisage the reason behind such behavior and speech [23]. Practitioners support the family members to recognize that knowledge of dementia is useful to understand the behavior of people with dementia and that the cognitive decline does not affect the identity of the person, only modifies their behavior.

### 3.3. Role of the Practitioners

During this rehabilitation support, practitioners do not teach any methods or provide instructions. It is the person with dementia and his/her family that make their own decisions on daily living. The practitioner strengthens their thoughts and choices by attaching positive meanings and urging them to be conscious of the co-beneficial and gratifying relationships. In daily living, families often complain about deterioration of cognitive function of people with dementia and difficulty in communication. These complaints can be used for improvement, because the families are aware of the situations to be tackled. Thus, practitioners can offer different perspectives and various ways to cope with the difficulties, until the people with dementia and families find their way to communicate with each other. In this way, it is the people with dementia and their families who take the initiatives in their own lives and make decisions independently, thereby gaining confidence to cope with the difficulties in the future. 

### 3.4. Characteristics of the Rehabilitation Program

The process of intervention is as follows: (1) selecting activities for collaboration; (2) sharing details of the current situation including declined capacities; (3) enhancing cognitive empathy through dementia education; (4) designing the intervention measures together; and (5) practice and feedback. The details of the process are described later in the chapter.

This program is characterized by the following 4 points: first, a holistic perspective is adopted, focusing on the “person,” and not on specific symptoms; second, according to the basic concept of OT, the therapy does not provide predefined activity tasks; third, the therapy is conducted in a tailor-made manner; and last, the practitioner is expected to empower people with dementia and their family members with a collaborative self-management attitude.

With respect to the first characteristic, a person-centered approach is recognized as the basic principle of the approach for dementia [24]. The key concepts are treating the person with dignity and respect, understanding his/her history and preferences, considering the perspective of the person, providing opportunities for the person to have social relationships and ensuring the person has the opportunity to attempt new activities or participate in activities they enjoy [25].

In regard to the second and third characteristics of pre-defined versus tailored approaches, non-pharmacological therapies for dementia including rehabilitation previously provided predetermined programs, especially in research [16,26] and there have been extensive discussions regarding the therapy (e.g., music therapy and reminiscence) that is effective for specific symptoms. However, systematic reviews have reported that those therapies have insufficient evidence [27,28,29,30]. This may be due to reductional thinking of the pharmacological approach to analyze and identify the symptoms and prescribe the appropriate medicines (in non-pharmacological therapy, prescribe pre-determined routine of activities, that is, selecting music therapy for depressive symptoms regardless of the preference of each person) [31]. Basically, OT recognizes activities as tools to improve function and/or to facilitate communication, based on the preference of each person. For example, if a person with dementia needs exercise of the lower limbs and he/she likes pop music, the practitioner may devise exercises for the lower limbs in tune with pop music. For communication, collaborative activities facilitate mutual understanding without directly considering the psychological aspects. This is the basic idea of OT. In practice, practitioners provide holistic person-centered tailor-made therapies for individuals involved in the activity and currently, the tailor-made approach is becoming the center of non-pharmacological treatment research for people with dementia to bridge the research-practice gap [16]. 

There exist two significant studies of tailor-made methods for people with dementia: “Individual goal-oriented cognitive rehabilitation (GCR)” [32,33,34,35] and TAP [18,19,20,21]. GCR focuses on cognitive functions in individuals with early-stage ADD. Goals are selected collaboratively in everyday settings to manage the difficulties most relevant for the person with dementia and his/her family members or supporters. As the goals are highly individualized, the intervention should be inevitably tailor-made. Intervention uses the compensatory and/or restorative approach and involves family members or other supporters where available. It is reported that collaborative intervention addressing individually-relevant goals can enhance not only everyday functioning but also QOL of the person with dementia and his/her family members. 

GCR focuses on cognitive function, while TAP focuses on dementia-related behavioral symptoms and functional dependence, which may result in poor QOL for people with dementia and their caregivers. The purpose of intervention is to reduce behavioral symptoms, functional dependence and caregiver burden. In the TAP intervention, which is a home-based occupational therapy intervention for people with dementia, the practitioner identifies the interests and capabilities of each person with dementia, develops and tailors activities to suit individual profiles and trains families in using activities as a part of their daily care routines. The use of TAP is not limited to the persons with the early-stage dementia, while GCR and the intervention of the present study focus on the early stage of dementia.

As above, TAP focuses on managing behavioral symptoms of dementia, whereas the intervention of the present study is focused ensuring that people with dementia have a self-management attitude to maintain a good relationship with others including their family members (the fourth point). Self-management of chronic disease is becoming more common as an approach for disease management such as diabetes, with currently no cure or treatment, only alleviation of symptoms. Self-management of people with dementia is also proposed, aiming to retain independence for as long as possible, by devising strategies to cope with symptoms based on an understanding of the disease [36,37]. This type of self-management would be limited to only the earliest stages of the disease and independence would eventually become impossible. This program focused on the self-management attitude to accept the present functioning and maintain a proactive attitude to retain social relationships including emotional management.

## 4. Intervention Process

Before initiation, it is important to confirm and disclose the purpose of the intervention with the person with dementia, his/her family member(s) and the practitioner: the aim of the intervention for people with dementia and their family members is to acquire a collaborative self-management attitude to cope with dementia. 

Furthermore, this intervention assumes the participation of people with early-stage dementia (not limited to ADD). Alzheimer’s Disease International mentions the importance of early diagnosis, “Maximize your quality of life,” “Plan for the future” and “Explain to your family, friends and colleagues what has changed in your life” [38]. The early stage is an important time for both the person with dementia and his/her family members to accept the changes due to dementia and to achieve a collaboration. Furthermore, self-monitoring becomes difficult for those in the intermediate and advanced stages of dementia [7,8,9]. Thus, it is all the more important to review the relationship with the family members for acceptance of changes due to dementia, in the early stages. The interventions are required to be conducted 10 times, once a week. It usually takes time for people with dementia and their family members to accept the changes due to dementia and build a positive mental attitude; however, this is highly dependent on the individuals involved.

Step 1: Choosing activities for collaboration

Based on preference and the living situation, the person with dementia and their families choose the activities that they can enjoy together in everyday lives without difficulty. The type of activities does not matter; choosing activities that the person with dementia and his/her family can continue, including domestic work, farming, hobby and exercise, because the purpose is to reconsider and reconstruct the relationship through collaboration and the chosen activity is “a communication tool.” Furthermore, as it becomes difficult for an individual to function independently as dementia progresses, it is important to choose the activity that can be performed in collaboration with families and carers.

One of the clinical cases of the author involved a person with dementia and his family selected bread making as the activity; the person with dementia (male) was a chemical engineer and bread making has processes in common with chemistry experiments.

Step 2: Sharing present situations including declined capacities

In the above case, the wife misunderstood that the person with dementia was capable of making bread alone or to take an initiative in the collaborative activity. This led to arguments as the wife urged the person with dementia to take the initiative, whereas he was not able to manage the situation. Therefore, she was slightly shocked to realize that she needs to lead him. Thus, when there is miscommunication or a discrepancy in mutual understanding, intervention is required; knowledge about dementia can help them restore the relationship. The practitioner analyzed the person’s ability and the process of the activity and advised them on the part that was difficult for the person of dementia and the method of collaborative support required.

As in the above case, the collaboration highlights the difficulties of the person with dementia and his current capabilities. Collectively handling a situation with the person with dementia, recognizing and adjusting with the deficits is the starting point for designing future lives-with- dementia. Psychological support should be provided as it is often difficult to face the reality, not only for the person with dementia but also for their families. For the person with dementia, such support can be effective for acceptance of the decline of his/her abilities and to appreciate the support from others, instead of lamenting over the decline and refusing support from others. As they continue to maintain the collaborative activity, it is expected that they will start to cooperate to cope with difficulties on other situations of daily living. Such generalization is one of the aims of this rehabilitation support.

Step 3: Enhancing cognitive empathy through dementia education

For families, dementia education can be informative for enhancing the cognitive empathy. Knowledge about dementia helps them to understand the behavior of the person with dementia. Due to cognitive decline, situation recognition can sometimes be difficult for the person with dementia and sometimes, subjective reality for the person with dementia is different from objective reality. Instead of correcting the recognition of a person with dementia, it can be effective for the families to understand how the cognitive decline can cause distorted recognition, in order to make a shared decision.

In the intervention, the practitioners assess the conversation between the person with dementia and his/her family members and provide appropriate advice, and/or attempt to create awareness. The issues are communicated in a manner that can be comprehended and anticipated by the people with dementia. Assuring anticipation is important, because it is difficult for the person with dementia to respond flexibly in response to external stimulus.

The following process should be used by the practitioner during verbal communication with a person with dementia:

1. First, listen without interrupting.
2. Then, confirm the person’s intended meaning by conversing with them.
3. Supplement the missing parts, if necessary and in such situations, use the words that the person with dementia has used, without paraphrasing.
4. Confirm the comprehended meaning step by step and reiterate the understood idea (this is the attitude of cognitive empathy).

It is important that people with dementia take the initiative in conversations. It is difficult for them to react flexibly to situations they have not expected, whereas it is rather easier for them to maintain the conversation within their expectations and comprehension. The most important attitude in the conversation is to add positive meaning to what the person with dementia has said and it is also recommended for the persons with dementia and their family members to try and laugh together about misunderstandings, since humor is important. (But be sure to laugh at mutual misunderstanding and not at the person) [39].

When asking questions to people with dementia,

1. Present alternatives, as open questions are difficult for them to configure answers.
2. Questions should be divided into small steps and the session should proceed after confirming the steps. Furthermore, it is desirable to obtain feedback and emphasize the good points.

Step 4: Devising collaborative measures to tackle dementia

In this process, it is important for the person with dementia and families to learn that every behavior is multifaceted and finding positive aspects and interpretations is possible in any kind of behavior. For example, in the bread-making case mentioned above, the person with dementia and his wife found a way to work complementarily in a cooperative activity. Even though he might gradually lose his abilities, the bread-making activity can be continued by devising ways of assisting him. Continuation of collaborative activities help in cultivating a habit of discussing and managing the difficulties together and it can be expected that collaboration will also be continued in daily living. As another example in daily living, the families often complain of the behavior of repeatedly confirming the schedule by the person with dementia. Memory decline and anxiety can be the background factors for such behavior but as a positive aspect, the person with dementia is willing to follow a schedule, keep promises and looks forward to the appointment. The practitioners can offer advice to collaboratively devise measures to remember the schedule, instead of trying to manage and control the behavior against the will of the person with dementia. In the above example, the method of writing daily schedule on a white board is often attempted. In order for such strategies to take root in everyday life, “fine adjustment” is necessary and it might be effective to carry out the task repeatedly by cooperation of the person with dementia and the family in piling small ingenuity, such as contents to write, how to write the time, size of letters and place to put the board. If necessary, the practitioner joins the collaborative work, gives advice and demonstrates ways to support, however, even in this situation, it is important to device a tailor-made way to fit their ways of living, not to force pre-decided ways as the medically reasonable way is not always the most appropriate way for their life style [16]. While collaborating with each other, the person and the family become explicitly aware of the relationship. This is the process to highlight the residual functions and strengths of the person with dementia. Through that experience of cooperation, they can learn to pay attention to the residual function and make use of their strengths. It may take time but it is expected that they will find mutual benefits in the process of collaboration.

Step 5: Practice and feedback

**Figure 1:**
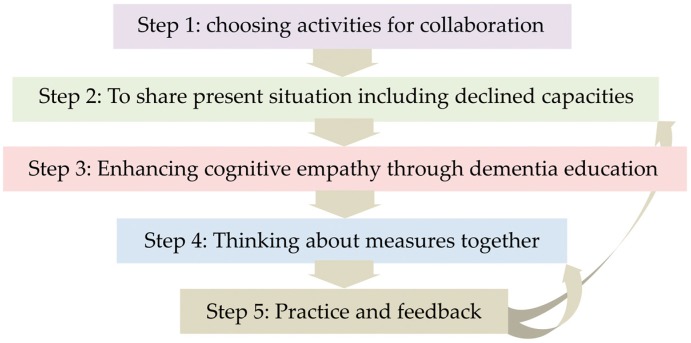
Intervention process is a trail-and-error procedure.

The practitioner gives advice on the collaborative work and challenges in daily life and strengthens their positive thoughts. For example, regarding the adopted measure of writing on a white board, if the person with dementia develops the habit of checking the board instead of repeatedly enquiring, the practitioner strengthens the process of collaboration, rather than the change behavior of repeated enquiry. It is important to explicitly create awareness, to develop the confidence to deal with future challenges in daily living by collaborating together (Figure 1).

If the person with dementia and his/her family succeed to find benefits in the process of collaboration to cope with challenges, they may be able to lead meaningful lives-with-dementia. 

### Expected Effects

The goal of this intervention is that the person with dementia and his/her family acquire a self-management attitude in co-beneficial interdependent relationships. The person with dementia and their family are expected to develop the mindset to confront various challenges encountered during the disease progression. It may be expected for the people with dementia and their families to improve “Sense Of Coherence (SOC),” which refers to the dynamic feeling of confidence that ‘(i) the stimuli, derived from one’s internal and external environments in the course of living are structured, predictable and explicable; (ii) resources are available to one to meet the demands posed by these stimuli; and (iii) these demands are challenges, which are worthy of time investment and patient engagement’ [40]. These three elements reflect the components of comprehensibility, manageability and meaningfulness of SOC, which can be one of the factors influencing social health in people with dementia [11]. Abstract thinking becomes difficult for people with dementia and change in mindset is not clearly recognized by them, however, change in attitudes may be observed in daily living. For example, even if they fail, they may not hide the failures or panic, however, may portray a picture of having managed the situation along with others. Dependence during dementia may affect dignity, however, dignity can be retained with such interdependent relationships. It is also expected that social QOL of both people with dementia and their family members would be enhanced and the care burden is also expected to be reduced. 

The intervention is conducted in an individualized manner and the expected effects may differ according to the individuals, because of the differences in lifestyles and individuals. Support should be provided for each person with dementia and his/her family to find a way to manage their life with dementia according to their respective lifestyles and to improve their quality of living. 

Narratively, in the bread making case, the person with dementia realized after 10 sessions that he had lived according to the norm and standard that he had made, not on the standard of others and he had confidence to continue to live by his own standards in future. He may have realized the feeling of self-confidence, which would not be lost in the progression of disease. His wife also changed her perspective after the session. She had been concentrating on the deterioration of cognitive function, especially memory so far but she realized the fact that the identity of the person with dementia had not been changed and that it would not be changed in the course of the disease progression. She realized that she tended to focus on his residual functions. Earlier, she had been worried about the future when various symptoms would appear but later she believed that she would deal with all the situations. Furthermore, instead of trying to manage his behavior, she would prefer to adjust the environment so that he could demonstrate his residual abilities and that this attitude was also beneficial to her. Thus, she had accepted the person with dementia with his current capabilities.

## 5. Conclusions

The intervention is expected to empower people with dementia and their family members with a self-management attitude to live lives-with-dementia in collaboration and consider their lives worth living. 

Life-with-dementia involves a continuous process of coping with various challenges in daily living. A collaborative process of coping with the challenges may confer a sense of “well-being.” This model of rehabilitative support emphasizes the collaboration; thus, it is desirable to initiate this at an early stage, when the person with dementia can understand the situation and recognize the meaning of interdependence. There is no available cure for dementia; however, this rehabilitative support may help to provide a better QOL.

The model has a limitation owing to the lack of empirical evidence. Thus, in future studies, the effects should be verified to promote evidence-based rehabilitative practice for persons with dementia and their family members.
